# High Glucose Attenuates Shear-Induced Changes in Endothelial Hydraulic Conductivity by Degrading the Glycocalyx

**DOI:** 10.1371/journal.pone.0078954

**Published:** 2013-11-18

**Authors:** Sandra V. Lopez-Quintero, Limary M. Cancel, Alexis Pierides, David Antonetti, David C. Spray, John M. Tarbell

**Affiliations:** 1 Department of Biomedical Engineering, The City College of The City University of New York, New York, New York, United States of America; 2 Department of Molecular & Integrative Physiology, University of Michigan, Ann Arbor, Michigan, United States of America; 3 Dominick P. Purpura Department of Neuroscience, Albert Einstein College of Medicine, Yeshiva University, Bronx, New York, United States of America; University of Patras, Greece

## Abstract

Diabetes mellitus is a risk factor for cardiovascular disease; however, the mechanisms through which diabetes impairs homeostasis of the vasculature have not been completely elucidated. The endothelium interacts with circulating blood through the surface glycocalyx layer, which serves as a mechanosensor/transducer of fluid shear forces leading to biomolecular responses. Atherosclerosis localizes typically in regions of low or disturbed shear stress, but in diabetics, the distribution is more diffuse, suggesting that there is a fundamental difference in the way cells sense shear forces. In the present study, we examined the effect of hyperglycemia on mechanotranduction in bovine aortic endothelial cells (BAEC). After six days in high glucose media, we observed a decrease in heparan sulfate content coincident with a significant attenuation of the shear-induced hydraulic conductivity response, lower activation of eNOS after exposure to shear, and reduced cell alignment with shear stress. These studies are consistent with a diabetes-induced change to the glycocalyx altering endothelial response to shear stress that could affect the distribution of atherosclerotic plaques.

## Introduction

Hyperglycemia is a characteristic feature of type 1 and type 2 diabetes that has been long associated with both micro and macrovascular dysfunction [1]. Factors such as dyslipidemia, hyperinsulinemia, and hypertension together with endothelial dysfunction interact during the progression of the disease. Given the complex interplay among these factors the mechanisms that lead to vascular complications are not completely understood. However, it has been suggested that an impaired endothelium is an early marker of diabetes induced vascular pathology [2].

The interaction between blood flow and the endothelium is not only a result of soluble factors, but also includes the mechanosensing of shear forces that are exerted by blood flow [3]. This sensing enables the endothelium to strictly and acutely enforce vasoregulation through the production of vasodilators such as nitric oxide (NO) and prostacyclin (PGI_2_) and vasoconstrictors such as endothelin (ET) [4]. Shear forces also affect the endothelial transport barrier through regulation of apoptosis and mitosis rates that control the leaky junctions that influence LDL transport [5], [6] as well as through hydraulic conductivity (Lp) whose shear response is mediated by the glycocalyx (GCX) [7].

The GCX, which covers the surface of the endothelial cells, consists of proteoglycans linked either to the membrane (glypicans) or to the cytoskeleton (syndecans) with extracellular side chain glycosaminoglycans (GAGs) covalently linked to the core proteins. The most abundant of these GAGs are heparan sulfate (HS), chondroitin sulfate (CS) and hyaluronic acid (HA) [8], [9]. The GCX not only constitutes a sieve to prevent transvascular leakage of macromolecules [10] but also moderates hydraulic conductivity (Lp). In the last few years, evidence has emerged that damage to the GCX plays a pivotal role in several vascular pathologies. In vivo and in vitro experiments have shown that enzymatic depletion of HA leads to a reduction in thickness of the GCX, an increase in endothelial hydraulic conductivity [10], [11] and in the permeation of the GCX by macromolecules that can be reversed by infusing a mixture of hyaluronan and chondroitin sulfate [11].

The GCX may contribute as much as 60% of the hydraulic resistance of the capillary wall [12]. Loss of GCX leads to vascular abnormalities including increased permeability [7], [12], monocyte and platelet adhesion and impaired NO bioavailability [13]. Therefore one can conclude that the GCX acts as a protective structure to ensure homeostasis of the vasculature. By comparing the distribution of GCX permeable and impermeable tracers, Nieuwdorp et al. [13] demonstrated that the average GCX intravascular volume in healthy volunteers was nearly 2 liters and that hyperglycemic clamping elicited an almost 50% reduction in volume, coinciding with increased GCX components released in plasma.

Zuurbier et al., [14] demonstrated that 2 to 4 weeks of hyperglycemia in mice led to increased permeability of 70-kDa dextran but not 40-kDa dextran and suggested that hyperglycemia mainly affects the hyaluronan component of the GCX, altering the sieving function of this surface structure. In-vitro models have shown that in the presence of high glucose there is a significant shedding of the HS and the HA components into the culture media [15], and reduced levels of HS caused by hyperglycemia have been related to impaired alignment of endothelial cells exposed to shear stress [16].

The above studies provide evidence to support the hypothesis that hyperglycemia interferes with either the synthesis and or the shedding of at least two GAGs – HS and HA. However to our knowledge, there have been no studies that address whether hyperglycemia impairs the shear-induced Lp response. Previous in-vitro studies have established that shear stress increases endothelial hydraulic conductivity and that this effect can be attenuated by an eNOS inhibitor [17], demonstrating the relationship between endothelial Lp response and NO production. Further, several GAG components of the GCX affect the shear induced Lp response [7]. In vivo studies have supported a shear-induced Lp response as well. Using frog mesenteries and the modified Landis technique, Williams et al., demonstrated that certain vessels across the capillary bed altered filtration in response to changes in fluid shear stress and that this alteration was related to the magnitude of the stimulus [18]. According to their observation, arteriolar capillaries had no response, true capillaries had a moderate response and venular capillaries had a strong response to step changes in shear stress [19]. Further, Kim et al., demonstrated that this response could be eliminated by superfusion of a NOS inhibitor [20]. Therefore, in the present study, we have examined whether hyperglycemia can alter the endothelial response to shear stress, specifically related to Lp, NO production and cell alignment.

## Materials and Methods

### A. Chemicals and Materials

The following chemicals were obtained from Sigma-Aldrich Chemical Company (St. Louis, MO): bovine serum albumin (BSA-30% solution), Minimum Essential Medium Eagle (MEM), phenol red-free MEM (PF), penicillin-streptomycin solution, L-glutamine, trypsin-EDTA solution, HEPES, sodium bicarbonate, heparin (sodium salt, grade I-A, 181 USP units/mg), fibronectin, and (6R)-5,6,7,8-Tetrahydrobiopterin (BH4). Fetal bovine serum (FBS) was purchased from Hyclone Laboratories (Logan, UT). Transwell polyester filters (12-mm and 24- mm diameter, 0.4-µm pore size) were purchased from Costar (Cambridge, MA). Primary Anti- Heparan Sulfate 370255-1 was purchased from Amsbio (Lake Forest, CA), Biotinylated Hyaluronic Binding Protein 385911 was purchased from Calbiochem (Billerica, MA) and primary Anti-Glypican-1 66909 and Anti Syndecan-1 5632 were purchased from Santa Cruz (Dallas, Texas). The secondary antibodies IgM and IgG were purchased from Molecular probes (Carlsbad, CA) and the Western blot kit, from Cayman (Ann Arbor, MI). The Phospho-eNOS (Ser1177) and β-actin antibodies were obtained from Cell Signaling (Beverly, MA).

### B. Bovine Aortic Endothelial Cell Culture and insert preparation

BAEC were purchased from VEC Technologies (Rensselaer, NY) and from Lonza (Allendale, NJ) and grown in T-75 flasks with 10% FBS-MEM. The flasks were kept at 37°C in 5% CO_2_ balanced air. The model for the endothelium was developed by plating BAECs at a density of 6×10^4^ cells/cm^2^ on 12 and 24 mm diameter Transwell polyester filters previously coated with fibronectin. The filters were incubated for 6 days with either 5 mM glucose (NG) or 25 mM glucose (HG) 10%FBS-MEM. Cells were used from passages 5 to 10.

### C. Determination of the effect of High glucose on the Shear Stress induced Lp response

An apparatus developed in our lab [7], [12] to measure water flux across cell monolayers was kept inside a Plexiglas box maintained at 37°C. The media used during the experiment consisted of 1%BSA-MEM enriched with BH4 (60 µM), a cofactor for eNOS activity [21], [22].

The seeded 12 mm filters were placed inside a chamber to form a luminal (top) compartment and an abluminal (bottom) compartment separated only by the BAEC monolayer. The abluminal compartment was connected to a reservoir via Tygon and borosilicate glass tubing. The vertical displacement of the reservoir with respect to the liquid covering the cells, allowed us to apply a hydrostatic pressure differential across the monolayer. When a 10 cm H_2_O differential pressure was applied, the volumetric flow rate (Jv) was measured by tracking the position of a bubble that was inserted into the calibrated borosilicate glass tube. The hydraulic conductivity was calculated from the relationship: Lp  =  (Jv/A)/ΔP, where A is the BAEC monolayer area and ΔP is the pressure differential across the monolayer. After 60 minutes of applied pressure differential to drive water flux, a baseline Lp was established, and a defined shear stress was applied to the endothelial monolayer using a rotating disk separated by a distance h (500 µm) from the monolayer surface. The rotating disk generated a fluid shear stress distribution on the monolayer surface defined by: *τ* = *μ*×*ω*×*r/h,* where *μ* is the viscosity of the media, *ω* is the rotational speed, and r is the radial distance from the center of the disk. The parameters were adjusted in order to achieve a maximum steady shear stress of 20 dyn/cm^2^ at the edge of the disk, and this is the value that is reported in the tables and figures. The average shear stress over the entire filter area is 2/3 of the maximum. During the experiment, the luminal compartment and the reservoir were supplied with gas (5% CO_2_-95% balanced air) to maintain the experimental media at the physiological pH of 7.4. The Lp values were recorded for a total of four hours.

Two experiments were always run side-by-side. One endothelial monolayer was grown in normal glucose, while the companion monolayer was grown in high glucose. These pairs of monolayers originated from the same flask and passage and were grown for six days in the same tray.

### D. Heparan Sulfate, Hyaluronic Acid, Syndecan-1 and Glypican-1 Determination

Monolayers were grown in either NG or HG for six days. For immunocytochemistry, cells were rinsed with cold PBS and fixed with 4% paraformaldehyde-PBS for 15 minutes, blocked with 4%BSA-PBS for one hour, incubated with either of the following primary antibodies: Heparan Sulfate, Hyaluronic Acid, Syndecan-1 and Glypican-1diluted 1∶50 in 4% BSA-PBS for 3 days and washed gently with 1% BSA-PBS. The monolayers were incubated with secondary antibody for 1 hour: Alexa Fluor 488 conjugated with anti-mouse IgM or anti-biotin or anti-rabbit IgG diluted 1∶500. The monolayers then were washed 5 times and at least 4 random fluorescent images where taken per condition with the LSM 510 microscope manufactured by Carl Zeiss Inc. The intensity of the images was quantified with the software image J.

### E. Shear Induced Alignment Determination

BAEC monolayers were grown on 24 mm diameter inserts for six days in either NG or HG, then the media was replaced with 5% FBS- 5%BSA MEM and the inserts were placed inside the compartment of an apparatus that was designed to hold a 6 well tray with inserts. A rotating disk above each insert generated a fluid shear stress distribution on the monolayer surface as described above. All 6 disks with shafts were driven by a humidity resistant stepper motor and controller (Anaheim Automation, Anaheim, CA). The apparatus holding the inserts was placed inside the incubator to control for temperature and pH, and the monolayers were sheared for 24 hours at a maximum level of 12 dyne/cm2. At the end of the experiments the inserts were removed and phase contrast photographs taken at randomly selected locations (n = 4) using the Nikon Eclipse TE2000-E inverted microscope. These fields were all located toward the outer edge of the rotating disk where the shear stress was maximal. The length, width and angle of alignment of the cells in the field were measured with image J. The results were reported as the aspect ratio and alignment angle. A total of 24 fields were chosen for each condition (HG vs. NG).

### F. Shear Experiments to determine eNOS activation

BAEC monolayers grown in either NG or HG were placed inside the 6 well shearing apparatus where they were sheared for 4 hours at a maximum level of 20 dyne/cm2. During this time, the apparatus with the cells as well as the static controls were maintained inside the incubator to control for temperature and pH. Then the cells were collected for western blots to quantify total and phosphorylated eNOS.

### G. Western Blotting for eNOs, Phospho-eNOS, Syndecan-1 and Glypican-1

The activation of eNOS was determined after shear stress was applied for 4 hours, the monolayers were washed with cold PBS and scraped from the filters with a plastic spatula in the presence of RIPA extraction buffer (1 mM NaHCO3, 2 mM PMSF, 1 mM Na3VO4, 5 mM EDTA, 10% protease and phosphatase inhibitor cocktail tablet and 1% Triton-X). Protein concentration was determined with the DCTM Protein Assay from BioRad (Hercules, CA) using the ND2000 spectrophotometer from NanoDrop (Wilmington, DE). Western blot was carried out by standard techniques, loading 20 μg of protein into the gel wells and incubating over night with antibodies to eNOS and phospho-eNOS (Serine 1177), and the constitutively expressed protein β-actin, all from Cell Signaling Technologies (Beverly, MA), followed by reaction with matched secondary HRP conjugated anti-rabbit and anti-mouse IgG antibodies from Molecular Probes (Carlsbad, CA). The blots were scanned with the System FX Pro imaging system from Carestream (Rochester, NY) and quantified with Image J software. The same general procedure was carried out to collect protein from static monolayers treated with normal and high glucose as well as Mannitol controls, placing 30 ug of protein into the gel wells, and incubating with primary antibodies for Syndecan-1 and Glypican-1 diluted at a ratio of 1∶500.

### H. Statistical Analysis

Lp values, Heparan Sulfate, Hyaluronic Acid, Syndecan-1, Glypican-1 fluorescence, eNOS, Syndecan-1, Glypican-1 protein expression and alignment measurements are presented as means ± SE. Tests for statistical significance of Lp values were conducted using a 2-way ANOVA function from Excel, and a post hoc analysis using the Tukey method. Western blots, immunostaining and alignment pictures were quantified and analyzed using the NIH image J software. In these cases, statistical significance was determined using a 2-tailed t-test. For all of the experiments, *P*<0.05 was considered significant.

## Results

### A. Shear Stress Induced Lp response

**Figure 1:**
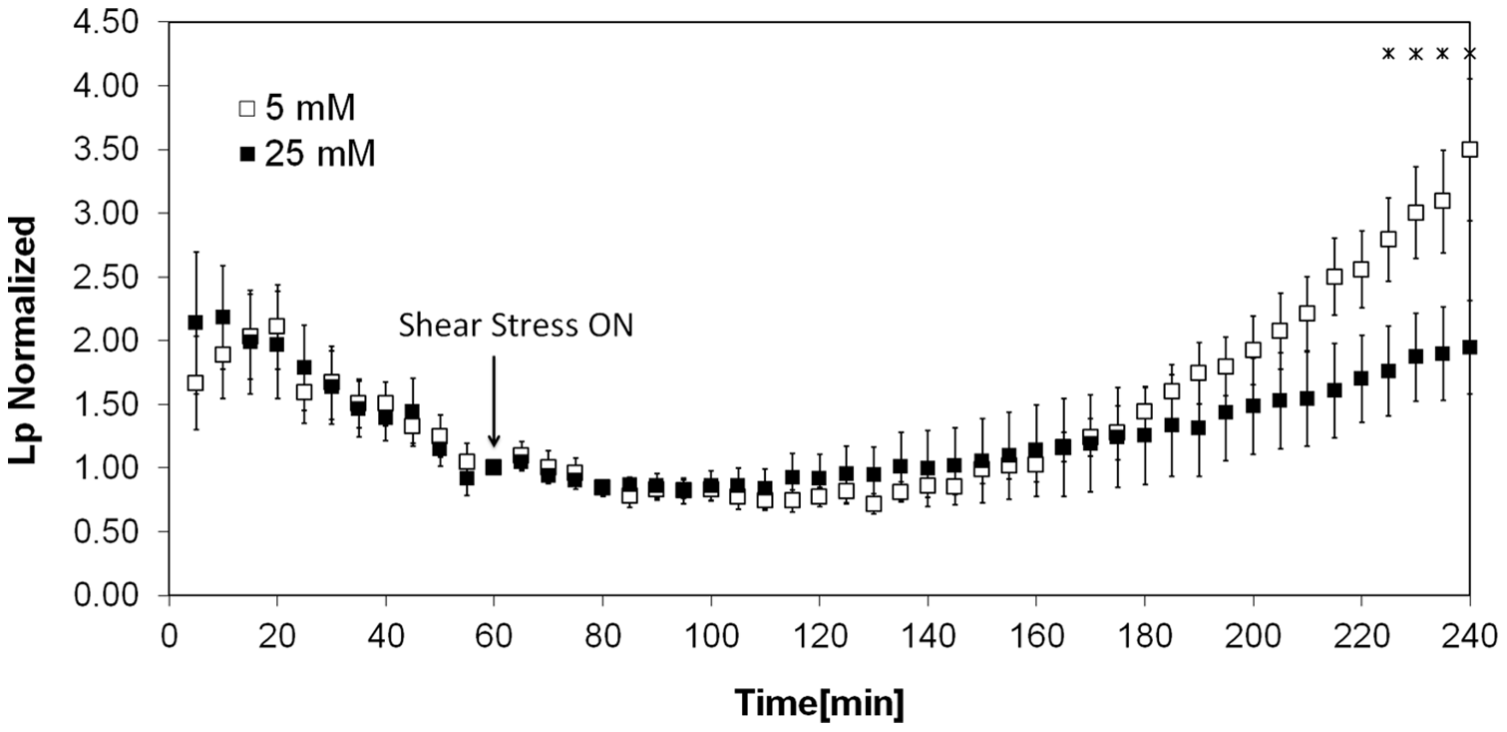
Normalized shear induced Lp response of BAEC monolayers grown in 5 At time 0_2_O was applied to the monolayers to drive water flow across them, and a baseline level was established after one hour. The Lp values were normalized to the baseline level for each monolayer. At time 60 min, a shear stress of 20 dyn/cm^2^ was applied and Lp recorded for three more hours. The monolayers that were treated with high glucose displayed a significant attenuation of the shear stress effect on Lp compared to controls (n = 6). The asterisks denote a significant differences between high glucose and control monolayers (p<0.05).

Figure 1 shows the shear stress induced Lp response for monolayers grown in high glucose media (25 mM) for six days compared to the companion control monolayers grown in normal glucose media. Both experiments showed the characteristic “sealing effect” where a gradual and significant reduction in Lp is observed during the 60 minutes following the application of the pressure differential [23]. The stable value at this point is considered as the baseline value. The baseline values for Lp (at 10 cm H_2_O pressure differential) for normal vs. high glucose were not significantly different from one another (3.82×10^−7^±0.57 cm/s/cm H_2_O vs. 4.71×10^−7^±0.65 cm/s/cm H_2_O, p = 0.34). Both values of the baseline Lp were within the normal ranges that have been reported for BAECs in previous studies [20], [24], [25]. As shown in the figure, the Lp values at different time points were normalized by the baseline value (value at 60 min) for both cases.

High glucose did not affect the “sealing effect” but significantly attenuated the shear induced Lp response. A similar behavior was observed for monolayers treated with heparinase [7].

### B. Glycocalyx immunostaining

**Figure 2:**
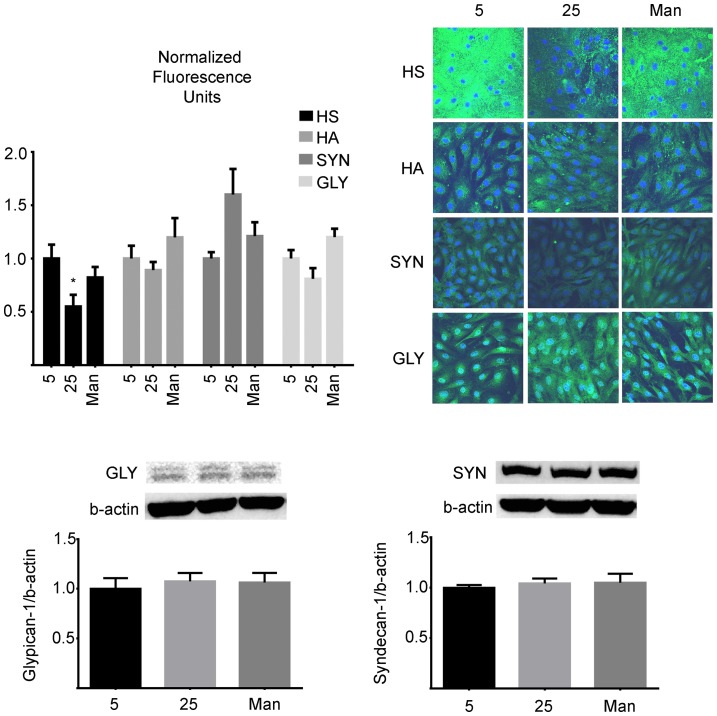
Glycocalyx of BAEC monolayers cultured in 5 BAEC monolayers were cultured in either normal (5 mM), high (25 mM) glucose or Mannitol control (25 mM) media. After six days, they were immuno-stained with antibodies against Heparan Sulfate (HS), hyaluronic acid (HA), syndecan-1 (SYN) and glypican-1 (GLY) as described in the methods. The monolayers were observed under a confocal microscope and four random fields were chosen for each condition. Only the mean fluorescence representative of Heparan Sulfate was significantly decreased to 55.5±11% in HG (p = 0.023). In addition, the contents of the core proteins Syndecan-1 and Glypican-1 were determined by western blot and consistenly showed that neither high glucose nor Mannitol had an effect on these components. (p-value >0.05, n = 4).

The visualization of Heparan Sulfate, Hyaluronic Acid, Syndecan-1 and Glypican-1 in the GCX was performed carefully, and it was necessary to fix the cells to preserve structural integrity. As shown in figure 2, incubating the cells with high glucose media (25 mM) for six days significantly reduced the presence of Heparan Sulfate to 55.5±11% of control (5 mM) (p = 0.023) while Hyaluronic Acid, Syndecan-1 and Glypican-1 were unchanged. In addition, as shown in figure 2, consistent results were obtained from western blots for the core proteins Syndecan-1 and Glypican-1.

### C. eNOS phosphorylation

**Figure 3:**
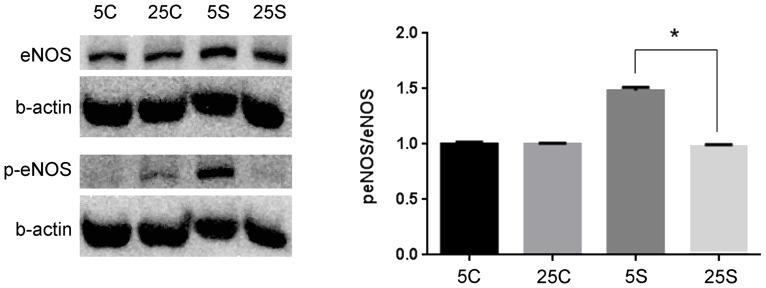
Representative Western blots for p-eNOS, eNOS and β-actin of BAEC monolayers. BAEC monolayers cultured in either 5 or 25(sheared monolayers over static controls values) of eNOS was significantly decreased from 1.48±0.01 to 0.98±0.03, (p = 0.0003, n = 3) when cells subjected to a maximum shear stress of 20 dyne/cm^2^ for 4 hours were incubated with high glucose compared to normal glucose. Baseline eNOS levels where not significantly changed (p = 0.15, n = 3) when cells were cultured in high glucose. Labels are as follows: 5c: 5 mM glucose static control, 25c: 25 mM static control, 5s: 5 mM with shear stress, 25s: 25 mM with shear stress.

For the Western blots the β-actin band served as a loading control and the ratio of p-eNOS to eNOS was obtained after normalizing the levels of each lane to each corresponding β-actin control. As shown in figure 3, the monolayers cultured in HG presented a lower phosphorylation ratio (sheared monolayers over static controls) than the monolayers cultured in normal glucose (0.98±0.01 vs. 1.48±0.03, n = 3, p = 0.02).

Additionally, the blots were re-probed with an antibody for total eNOS to make certain that a difference in phosphorylation was not a result of a change in the total eNOS protein. The total amount of eNOS protein did not differ between monolayers cultured in normal vs. high glucose (p = 0.15, n = 3).

### D. BAEC alignment upon Shear Stress

**Figure 4:**
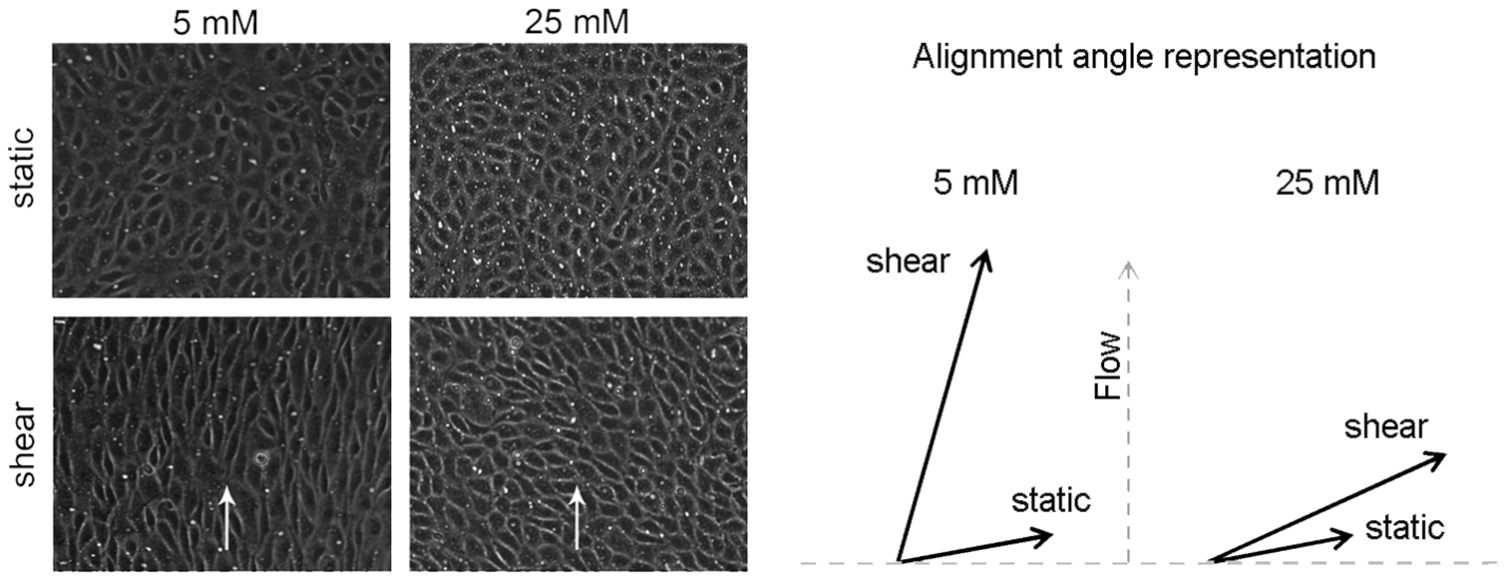
Representative alignment of monolayers cultured in high versus normal glucose subjected to shear stress. BAEC monolayers were exposed for 24/cm^2^, by using the 6 shaft apparatus described in the methods. The alignment of monolayers cultured in high glucose was determined by taking 6 random images of each insert (n = 3 inserts) in the near-edge region and measuring the aspect ratio and angle of each cell using the software image J. The aspect ratio and angle of alignment were used as indicators of alignment and were found to be significantly lower in monolayers cultured in high glucose versus monolayers cultured in normal glucose (aspect ratio: 1.98±0.09 vs. 3.61±0.11 and alignment angle: 85.24±1.24 vs. 20.92±0.65). Arrows on the picture indicate the direction of the flow.

Representative images of the alignment experiment are shown in figure 4. The aspect ratios and angles with respect to the horizontal axis used for characterizing alignment were calculated by dividing the length by the width of the cells, and by measuring the angle of the major axis in image J. After exposure to shear, the monolayers cultured in high glucose showed an average aspect ratio of 1.99±0.09, which was significantly lower (p = 0.0001) than the one for monolayers cultured in normal glucose 3.61±0.11 (n = 3). As shown in the figure, the direction of the flow was 90 degrees with respect to the horizontal and the alignment angle for cells cultured in normal glucose vs. high glucose was significantly different (85.24±1.24 vs. 20.92±0.65, n = 3). Our results are consistent with those of Yao et al. [26] and Ebong et al. [27] where it was shown that heparinase treatment of BAEC impaired cell alignment in the direction of 24-hour flow.

## Discussion

The observation that with diabetes atherosclerosis is distributed more uniformly in arteries, and not just limited to the typical atherogenic regions (regions of low shear or disturbed flow) near bifurcations and curvatures [28], and that flow-induced vasodilation is altered in patients with diabetes [29] suggests that there is something fundamentally different in the mechanosensing apparatus of the endothelium during the disease. These observations have motivated our study of the effects of hyperglycemia on mechanosensing.

To that end, we designed a study to determine if high glucose impairs the endothelial response to shear stress. Shear stress has been shown to regulate endothelial hydraulic conductivity by a mechanism that involves the transduction of mechanical forces into the production of NO [17], [20], [30] and to align endothelial cells in the shear direction [26].

The main observations of the present study were that the normal Lp increase caused by a step change in steady shear stress was significantly attenuated by high glucose (Figure 1), coincident with a decreased HS component (Figure 2), decreased eNOS activation (Figure 3) and decreased shear induced alignment (Figure 4). All of these observations are indicative of impaired mechanotransduction.

Previous studies have linked high glucose to dysfunction of the endothelium [31], [32], an organ whose main role is to sustain homeostasis of the vasculature in response to different stimuli, one of them being the shear stress caused by circulating blood. The GCX, which has been extensively described before [8], [9], [33], protrudes into the lumen with its GAG elements that bind plasma proteins and other solutes and are connected to core proteins linked either to the cell membrane or to the cytoskeleton. Because of its structure and location, the GCX is a prominent mechanosensor/transducer.

The GCX has received a great deal of attention for its role in mechanotransduction in recent years. Florian et al. [34] and Pahakis and Tarbell [8] showed that HS cleavage blocked shear induced NO production, and later Lopez et al. showed that HS cleavage also attenuated the shear induced Lp response [7], providing direct evidence for the role of HS in sensing and transducing flow induced forces. The present data demonstrate that there is an impaired endothelial Lp response to shear in high glucose, which is coincident with lower content of HS and reduced shear stress induced activation of eNOS.

The downregulation/degradation of HS by hyperglycemia could have important implications for diabetes. Several atheroprotective properties have been attributed to HS proteoglycans based on their capacity to inhibit monocyte binding to the subendothelial region [35], to inhibit arterial smooth muscle cell proliferation and to regulate fibroblast growth factor [36]. Our results in figure 2, which show decreased HS content in cells cultured in high glucose media are consistent with a previous study on BAEC and human endothelial cells, which showed that the HS proteoglycan perlecan is posttranslationally affected by high glucose allowing for less HS side chain binding [37]. The diminished HS content in high glucose media has also been explained by endothelial over-secretion of the heparanase molecule which specifically cleaves HS [38], and by the observation that in normal glucose levels, heparinase co-localizes with lysosomes preferentially around the nucleus but high glucose causes its dispersion towards the plasma membrane for subsequent secretion [15]. According to our findings it appears that only HS and not HA decreases significantly with high glucose, and that the core proteins Syncecan-1 and Glypican-1 are not key to the high glucose effects we have reported.

In human arteries, hyperlipidemia has been characterized by decreased HS [39] and diabetes has been shown to reduce the amount even more [40]. In a previous study, a 25% increase in baseline Lp was observed when BAEC were incubated with heparinase, the enzyme that selectively cleaves HS (HS reduction by 40%) [7]. While this difference did not reach statistical significance, it was very similar to the 23% increase in baseline observed in the present study when the same cells were incubated with high glucose (HS reduction by 45%). Degradation of HA with hyaluronidase (HA reduction by 45%) significantly increased baseline Lp [7], [8], but incubation with high glucose in the present study caused only an insignificant reduction of 12% that would not be expected to modify baseline Lp. However it is worth noticing that HA has been reported to be higher in serum of patients with diabetes due to increased levels of reactive oxygen species (ROS) able to stimulate hyaluronan degradation [41].

In other tissues, such as kidney [35] and retina [42], exposure to high glucose results in decreased synthesis of HS side chains and subsequent reduction of basement membrane anionic sites. Heparan sulfate therefore appears to play an important role in homeostasis of various tissues, and the studies discussed above suggest that high glucose may affect the synthesis and/or metabolism of HS proteoglycans and HS for various cell types. These data associate hyperglycemia induced modifications of GAGs and proteoglycans to atherosclerosis and kidney and retinal pathologies. Overall, shedding of the GCX is an important factor in atherosclerosis, because it exposes cell membrane adhesion molecules, such as vascular cell adhesion molecule-1 (VCAM-1) and intercellular adhesion molecule-1 (ICAM-1), which are required for the adhesion of leukocytes to the endothelial surface [43], therefore initiating an inflammatory response.

With respect to mechanosensing and adaptation, another in-vitro study showed that endothelial monolayers cultured in high glucose had reduced HS and were not able to align normally after being exposed to shear stress [16]. This observation and ours are in agreement with those of Yao et al. and Ebong et al. that show that HS is central in alignment of endothelial cells to 24 hour-flow [26], [27]. Because the core proteins Syndecan-1 and Glypican-1 are not affected by high glucose it is plausible to think that an agent that promotes the synthesis of Heparan Sulfate may be able to reverse the effects observed herein [44].

The shear induced Lp response, which is mediated by the production of NO [30] has not been observed before in a hyperglycemic environment. In the present study, the attenuated response that coincides with lower activation of eNOS suggests that hyperglycemia could affect the mechanotransducers upstream of NO and in fact we present evidence that shows a reduced amount of the HS component in hyperglycemic media (Figure 2).

Other studies have shown that phosphorylation of eNOS by several serine/threonine kinases is a fundamental step in NO production by endothelial cells. Phosphorylation by AMP kinase, Akt, (protein kinase A), or protein kinase B, on serine 1177, leads to increased activation of the enzyme and increased NO production in bovine cells [45]. Phosphorylation at serine 1177 is considered a marker of the activation state of eNOS in response to vascular endothelial growth factor (VEGF) and fluid shear stress. Certainly VEGF induced angiogenesis and hyperpermeability in vivo require eNOS derived NO [46]. The activation state of eNOS was examined with an antibody that recognizes serine 1177. As seen in figure 3, activated eNOS (p-eNOS) but not total eNOS content was reduced when the cells were incubated in high glucose and sheared for 12 hours. This observation is consistent with a previous study by Du et al. [47] that showed that hyperglycemia did not change the level of total eNOS, but reduced eNOS activation. Du and coworkers attributed this behavior to post translational modifications due to mitochondrial superoxide overproduction.

Another possibility for a reduced endothelial response is that once released, NO is quickly modified and its potency is reduced in a hyperglycemic environment due to the presence of advanced glycation end products which also modify eNOS [48]. NO is produced by endothelial cells from L-arginine in the presence of cofactors, the most common of them being tetrahydrobiopterin (BH4) [49]. In this context, Cai et al. [50] reported that during hyperglycemia BH4 is oxidized by peroxynitrite, which is formed by the interaction of superoxide with NO, resulting in an increase in the monomeric form of eNOS and hence an uncoupling of the enzyme. However it should be noted that our experiments used BH4 enriched media to ensure bioavailability of the cofactor.

Endothelial dysfunction (ED) explains an increased risk for cardiovascular disease in diabetic patients. Improved understanding of the mechanisms that lead to ED in this setting could provide new approaches for treatments. The mechanisms through which hyperglycemia causes ED are varied and may present complex interactions, however, the findings in this study point to the GCX as a possible target for early therapeutic intervention. A future approach for vascular disease may be based on the design and administration of copolymers [51] that are able to mimic the mechanosensing activity of an intact GCX, or the development of agents that can inhibit shedding of the GCX components [38] or that stimulate their synthesis in localized areas.
